# Attitudes and perceptions of Irish health care professionals regarding functional neurological disorder: A national survey

**DOI:** 10.1002/brb3.3362

**Published:** 2024-02-07

**Authors:** Roisin Vance, Sarah Clarke, Fiadhnait O'Keefe, Toni Galligan, Anne Doherty, Cora Flynn, Eric Kelleher, Aoife Laffan, Colin Doherty, Diane Gillan

**Affiliations:** ^1^ Physiotherapy Department Psychology Department Beaumont Hospital Dublin Ireland; ^2^ School of Physiotherapy School of Medicine Royal College of Surgeons Dublin Ireland; ^3^ Psychology Department St. Vincent University Hospital Dublin Ireland; ^4^ School of Psychology University College Dublin Dublin Ireland; ^5^ School of Medicine School of Psychology Trinity College Dublin Dublin Ireland; ^6^ Psychiatry Department Mater Hospital Dublin Ireland; ^7^ Nursing and Midwifery Department Health Service of Ireland Dublin Ireland; ^8^ Department of Psychiatry and Neurobehavioural science, School of Medicine University College Cork Cork Ireland; ^9^ Neurology Department St. James Hospital Dublin Ireland

**Keywords:** Functional Neurological Disorder, Perceptions, Survey, Attitudes, Health Care Professionals

## Abstract

**Background:**

Functional neurological disorder (FND) is a common and often disabling condition. Limited access to services for FND poses challenges both for patients and their health care providers. This survey explored the attitudes, experiences, support needs and training needs of health care professionals (HCPs) who provide care to individuals with FND in Ireland.

**Methods:**

A broad range of HCPs working with patients with FND in Ireland partook in an anonymous online 12‐item survey. Participants were recruited via professional bodies and snowball convenience sampling utilising social media and email invitation. Descriptive and inferential statistics were employed to analyze data.

**Results:**

A total of 314 HCPs working in Ireland completed the survey. 80% were female and over half worked in their current role for more than 10 years.   75% of the sample encountered three or less individuals with FND per month. Identified service‐related challenges to effective patient care included insufficient clinic time, lack of confidence explaining the diagnosis, and the need for greater access to specialist support.  Data revealed persisting negative attitudes toward FND patients among a proportion of respondents. The majority of respondents did not feel they received adequate education on FND, with the exception of neurologists, of whom 65% felt adequately trained.  The majority of respondents (85%) also felt that people with FND did not have access to appropriate FND services in Ireland.

**Conclusion:**

This study indicates that there is a significant need to improve FND education among HCPs in Ireland, in addition to developing appropriately resourced, integrated, multidisciplinary care pathways for the FND patient group.

## Graphical abstract

This survey explored the attitudes, experiences, support and training needs of health care professionals (HCPs) providing Functional Neurological Disorder (FND) patient care in Ireland. 314 HCPs completed the survey. Identified service‐related challenges included insufficient clinic time, lack of confidence explaining the diagnosis, and the need for greater access to specialist support. Data revealed some persisting negative attitudes towards FND patients and the majority of respondents did not feel they received adequate FND education. The need for enhanced education and development of appropriately‐resourced multidisciplinary FND care pathways was identified.

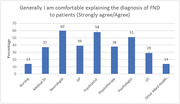

## 1 INTRODUCTION

Functional Neurological Disorder (FND) is a condition which arises primarily from a disorder of nervous system functioning, rather than identifiable pathophysiological disease (Stone et al., 2020).  Alterations in the functioning of key brain networks, rather than abnormalities in brain structure, underpin FND symptoms. The frequency and severity of FND symptoms can fluctuate (Hallett et al., 2022). While traditional aetiological models of FND were dominated by the psychodynamic concept of conversion of emotional distress or trauma into physical symptoms, it is now recognized that there is no one single causative mechanism for FND (Fobian & Elliott, 2019), and diagnosis by *DSM‐5* no longer requires identifying precipitating stressors, because these are not always found or readily identifiable (Perjoc et al., 2023). More recent psychological theories have focused more on how FND symptoms are produced rather than on why they may develop (Espay et al., 2018).

FND symptoms often follow a chronic course, causing high levels of disability and distress (Carson et al., 2011).  It is estimated that FND is the second most common neurological diagnosis after headache, with FND representing a significant proportion of new referrals to neurology clinics (Stone et al., 2010).

Despite its common presentation, both healthcare professionals (HCPs) and patients have reported negative experiences in relation to the diagnosis and management of FND. From the patient perspective, those with FND experience significant delays in diagnosis with often multiple admissions to hospital, multiple specialist reviews, unnecessary investigations and possible exposure to iatrogenic harm before a diagnosis is reached (Espay et al., 2018).  As a result, healthcare costs for FND have been shown internationally to be substantial (Stephen et al., 2021). Patients with FND often report delayed and poor communication in diagnosis, negative relationships with HCPs and challenges in accessing services and supports (O'Keeffe et al., 2021; Rawlings & Reuber, 2016). The patient experience can often involve feelings of stigma and a sense of invalidation regarding symptoms (Foley et al., 2022).

From the HCP perspective, clinicians describe challenges in the provision of appropriate care for patients with FND (Begley et al., 2023).  Barnett and colleagues reported that HCPs often felt uncertain about how best to manage patients with FND, and tended to refer to other disciplines, which was associated with less effective treatment, and a lack of clear communication (Barnett et al., 2022).   Warner and colleagues reported that doctors felt organisational barriers, such as lack of continuity of care and time pressures, were preventing effective management of patients with medically unexplained symptoms (Warner et al., 2017).  Some clinicians view FND symptoms as at least partially voluntary, feigned and/or deliberate (Aatti et al., 2016; Ahern et al., 2009; Kanaan et al., 2011; Tinazzi et al., 2022; Yogarajah et al., 2018), and associate it with conditions such as factitious disorder and malingering. This lack of essential understanding that FND is a separate and unrelated condition where patients are not feigning symptoms can lead to further negative interactions.

These difficult experiences can result in significant additional distress and contribute to chronicity of disability, as patients seek further clinical opinions and more supportive interactions (Rawlings & Reuber, 2016). Such factors, combined with the variable prognosis associated with FND (Gelauff et al., 2014; Kanaan et al., 2011) can cause challenging interactions for both patients and HCPs.

Despite the high incidence of FND and the known benefits of a multidisciplinary model of care (Perez et al., 2021), FND remains a condition for which services in both acute and community settings in many countries remain extremely limited (Ducroizet et al., 2023).  Lack of undergraduate and postgraduate education and a lack of training and knowledge regarding management of FND has been identified as a barrier across HCP disciplines (Begley et al., 2023; Lehn et al., 2019).

Most studies to date exploring HCP's attitudes to FND have focused on medical professions, and there has been little exploration of the views of other professionals who contribute to multidisciplinary care.  This gap in research is pertinent considering the increasing value placed on interdisciplinary working, and the level of clinical responsibility taken by members of the multidisciplinary team (MDT) (Bennett et al., 2021).

This study aimed to explore attitudes and perceptions of a broad range of HCPs who provide care for individuals with FND in Ireland.  The study aimed to identify the barriers and challenges and potential facilitators currently experienced by those providing FND care.  This research aimed to inform national policy on service development and design, and offer recommendations for increasing awareness and education among HCPs regarding FND.

## 2 METHOD

### 2.1 Survey design and development

A 12‐item questionnaire was developed, adapted from a survey developed by Lehn et al. (2019). The selected items aimed to capture three key themes: ***Challenges to working with FND*** (e.g., “I generally feel well supported by other health professionals who refer patients with FND to me”); ***Attitudes toward patients with FND*** (e.g., “These patients’ symptoms are real”); ***Knowledge and Training in relation to FND*** (e.g.,“I received adequate education about FND as part of my training or CPD”). Each item required a 5‐point Likert‐scale response, where 1 = “Strongly Agree”; 2 = “Agree”; 3 = “Neither Agree nor Disagree”; 4 = “Disagree”; and 5 = “Strongly Disagree.” Two additional open‐ended questions were included. One asked which specialisms HCP respondents thought were most appropriate for managing FND. There was also a free‐text option for HCP respondents to add further comments.

Participants were also asked to provide demographic information (i.e., gender, age) and professional experience and work setting (profession, years of professional experience, health care setting) (Appendix A).  See Appendix B for complete list of survey questions. The survey was developed and administered online using Qualtrics software.

Ethical approval was obtained from Trinity College Dublin, School of Medicine Research Ethics Committee (TCD SOM REC No: 20210207).

### 2.2 Participants

Inclusion criteria for participation included HCPs (>18 years), who work with patients with FND, working in the fields of neurology, psychiatry, psychology, general practice, nursing, physiotherapy, occupational therapy, speech and language therapy or any health care worker in another clinical area related to FND. Exclusion criteria were HCPs who were retired, working outside of the Republic of Ireland or who did not have any clinical contact with FND patients.

### 2.3 Procedure

HCPs were invited to participate in the survey via several methods between October 2021 and January 2022. A survey advertisement including QR code and online link was produced and disseminated to appropriate professional bodies described below via email. 

1. Email blasts occurred between October and December 2021 with the Qualtrics survey link sent to mailing lists from established national professional representative bodies, including Irish Society of Physiotherapy, The Psychological Society of Ireland Divisions of Clinical, Counselling and Neuropsychology, The Association of Occupational Therapy Ireland, The Irish Society of Speech and Language Therapists, Royal College of Physicians, Royal College of Surgeons, Irish Institute of Clinical Neurosciences, Irish Association for Emergency Medicine, The College of Psychiatrists of Ireland, Irish College of General Practitioners, and the Nursing and Midwifery Board of Ireland.
2. Circulation through snowballing convenient sampling also occurred where members of the *National FND Special Interest Group* were asked to circulate to their colleagues in their networks and teams, who met inclusion criteria.
3. A link to the survey was circulated via social media Twitter on 2/11/21. The survey link was closed on January 31, 2022.

Opening the link led participants to read the information leaflet and provide consent to participate and proceed to the survey. The time required to complete the survey was estimated at 3–5 min. All responses were anonymised, with no identifying information gathered.

### 2.4 Data analysis

Data analyses were conducted using IBM Statistics 27 (Statistical Package for Social Sciences, SPSS version 27.0). Descriptive analyses were used to compare frequencies across demographic variables and questionnaire items. Demographic and clinical data were stratified into categorical variables with two or more groups in each variable, for example, number of patients seen (<1 or >1 patient per month). Healthcare settings comprised hospital and community setting (i.e., community mental health service, disability services, rehabilitation services, private practice, voluntary organisations, and older adult services). Percentage or frequency of agreement among participants was evaluated by those who endorsed either “Agree” or “Strongly Agree.” Similarly, rates of disagreement were captured by those who endorsed either “Disagree” or “Strongly Disagree,” in keeping with Lehn and colleagues (Bennett et al., 2021).

## 3 RESULTS

A total of 314 HCPs working in Ireland completed the survey. Table 1 summarizes the demographic and professional characteristics of the respondents. The majority of respondents (80%) were female and aged between 36 and 45 years (38.9%). Over half of the sample (63%) have worked in their current role for 11 years or more. In terms of professional background, the largest category of respondents (23.9%) was physiotherapists, followed by medical doctors (22%). 15.6% of respondents were nurses, and the remaining respondents (38.5%) were allied health professionals other than physiotherapy, including psychologists, occupational therapists, speech and language therapists, dieticians, audiologists, and social workers. Almost three‐quarters of the sample were working in a hospital setting.

**TABLE 1:** Demographic and professional characteristics of sample (*n* = 314).

	Frequency (% total sample)
**Gender**	
Female	252 (80.3%)
Male	61 (19.4%)
Other	1 (0.3%)
**Age**	
18–25	7 (2.2%)
26–35	94 (29.9%)
36–45	122 (38.9%)
46–55	69 (22%)
55–65	18 (5.7%)
>65	4 (1.3%)
**Profession**	
Nursing	49 (15.6%)
Medical professionals (total)	69 (22%)
Medical doctor (specialism unspecified)	19 (6.1%)
Neurologist	20 (6.4%)
GP	18 (5.7%)
Psychiatrist	12 (3.8%)
Physiotherapist	75 (23.9%)
Psychologist	55 (17.5%)
Occupational therapist	47 (15%)
Other allied health professional*	11 (3.5%)
Missing data	8 (2.5%)
**Years employed in current profession**	
<5 years	48 (15.3%)
6–10 years	67 (21.4%)
11–15 years	81 (25.9%)
16–20 years	52 (16.6%)
>21 years	65 (20.8%)
**Service setting**	
Hospital	227 (72.5%)
Primary care	27 (8.6%)
GP practice	17 (5.4%)
Other	42 (13.4%)

*Speech & language therapy *N* = 5; social work *N* = 2; audiology *N* = 2; dietetics *N* = 2.

**FIGURE 1:**
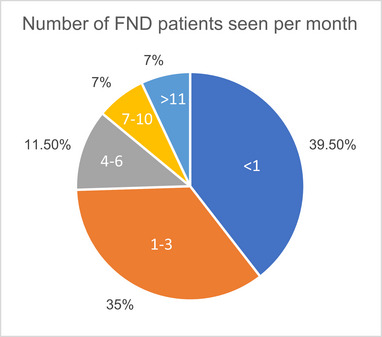
Number of FND patients seen per month by respondents.

Respondents indicated the number of patients with FND they saw clinically per month. 75% of the sample encountered three or fewer individuals with FND per month (see Figure 1).

### 3.1 Challenges to working with FND

Service‐related challenges to working with patients with FND were explored through six Likert‐scale items. 53% of respondents either agreed or strongly agreed with the statement *“I don't think I can help these patients much without specialist support*.” Free‐text responses provided additional information on the types of specialist support respondents felt would be appropriate, with the majority highlighting the need for multidisciplinary input for patients with FND.

Respondents were also asked about the amount of clinical time available to see patients with FND. 53% of respondents agreed/strongly agreed with the statement “*I don't have enough time to deal with these patients appropriately in my clinical setting*.” 74% of neurologists felt they had insufficient time in clinic for patients with FND, which is higher than the rate for the group as a whole. However, 77% of psychiatrists disagreed about time pressure in clinic.

Chi‐square analysis did not indicate any significant difference in the frequency of responses across the health care settings in terms of clinical time available to see patients with FND  (i.e., hospital vs. community setting; (*X* ^2^ (2, *N* = 243) = 0.875; *p* = .646).

Perceptions of peer support when working with patients with FND were explored with the following statement “*I generally feel well supported by other health professionals who refer patients with FND to me*.” A greater number of respondents disagreed/strongly disagreed with this statement than agreed/strongly agreed (see Table 2).

**TABLE 2:** Frequency of responses to survey items.

	Strongly Agree	Agree	Neither Agree/Disagree	Disagree	Strongly Disagree
***Challenges to working with FND***					
I don't think I can help these patients much without specialist support	13%	40%	19%	24%	4%
I don't have enough time to deal with these patients appropriately in my clinical setting	20%	33%	20%	23%	4%
I generally feel well supported by other health professionals who refer patients with FND to me	2%	23%	39%	30%	6%
People with FND have access to appropriate services to manage their FND in Ireland	2%	2%	11%	41%	44%
Generally I am comfortable explaining the diagnosis of FND to patients	8%	31%	15%	34%	12%
When I see patients with FND I often find that the referring doctor was not honest/ open with the patient	14%	32%	29%	23%	1%
***Attitudes toward patients with FND***					
I often find these patients demanding and difficult to deal with	10%	32%	26%	27%	5%
I am often worried that these patients are actually malingering, faking or feigning	2%	13%	17%	47%	22%
These patients’ symptoms are real	36%	52%	10%	2%	<1%
***Knowledge and training in relation to FND***					
I received adequate education about FND as part of my training or CPD	5%	6%	16%	37%	26%
I am interested in learning more about working with people with FND	49%	40%	8%	2%	1%

Overall, the majority of respondents (85%) disagreed/strongly disagreed with the statement “*People with FND have access to appropriate services to manage their FND in Ireland*.”

Additional free‐text responses also highlighted a lack of established care pathways from acute to community settings: *“We are lacking sufficient follow‐on services in Ireland from acute to community settings,”* resulting in inappropriate readmission to acute hospital settings: *“I see so many patients re‐admitted to acute hospital as intervention post‐discharge is so poor.”*

**FIGURE 2:**
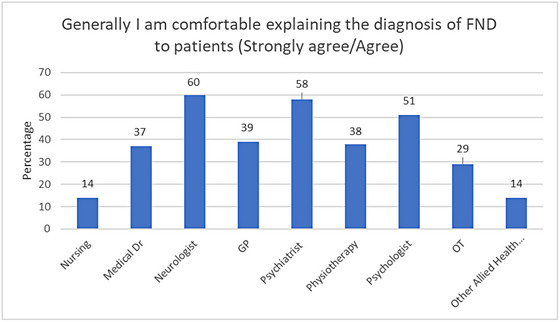
Rates of Confidence in explanation of FND diagnosis by profession.

Only 39% of respondents felt comfortable explaining the diagnosis of FND to patients. Neurologists and Psychiatrists had the highest rates of confidence in explaining a diagnosis of FND, with the lowest rates reported by nursing staff and those in the category of “Other” allied health professionals (see Figure 2).

There was a small trend between confidence in discussing a diagnosis of FND and levels of clinical contact with patients per month (i.e., less than one per month compared with more than one per month)  (*X* ^2^ (1, *N* = 249) = 5.39; *p* = .20); with a small effect size.

Almost half (47%) of the sample either agreed/strongly agreed with the statement “*When I see patients with FND I often find that the referring doctor was not honest/open with the patient*.”

Additional free‐text responses regarding delayed or unclear diagnosis highlighted the resultant impact on management and rehabilitation of FND: *“Often referral information does not state FND, which can lead to an incorrect management focus” ….“this makes the rehab process very difficult.”* Lack of consistency in relation to diagnostic terminology was also cited as a further challenge: “*The terminology used by different professionals can cause confusion.”*

### 3.2 Attitudes toward patients with FND

42% of respondents agreed/strongly agreed with the statement “I often find these patients demanding and difficult to deal with,” while 32% disagreed/strongly disagreed. There was no significant association between health care setting (i.e., acute hospital versus community) and perceptions of patients with FND being difficult or demanding to deal with (*X* ^2^ (1, *N* = 220) = 0.58; *p* = .447).  Frequency of clinical contact with FND patients was also not significantly associated with this particular variable  (*X* ^2^ (1, *N* = 220) = 1.85; *p* = .174).

A majority (69%) of respondents disagreed or strongly disagreed with the statement “I am often worried that these patients are actually malingering, faking or feigning.” However, 17% of respondents neither agreed or disagreed with this statement. 10% of the sample neither agreed nor disagreed with the statement “These patients’ symptoms are real.” There was no significant association between perceptions about symptom validity and frequency of clinical input with FND patients (*X* ^2^ (1, *N* = 241) = 0.84; *p* = .773).

Free‐text responses provided additional information in relation to negative attitudes toward patients with FND: “In Ireland I find the approach to FND is significantly worse with more of an attitude of ‘how can I get the patient out of my clinic?’”

### 3.3 Knowledge and training in relation to FND

**FIGURE 3:**
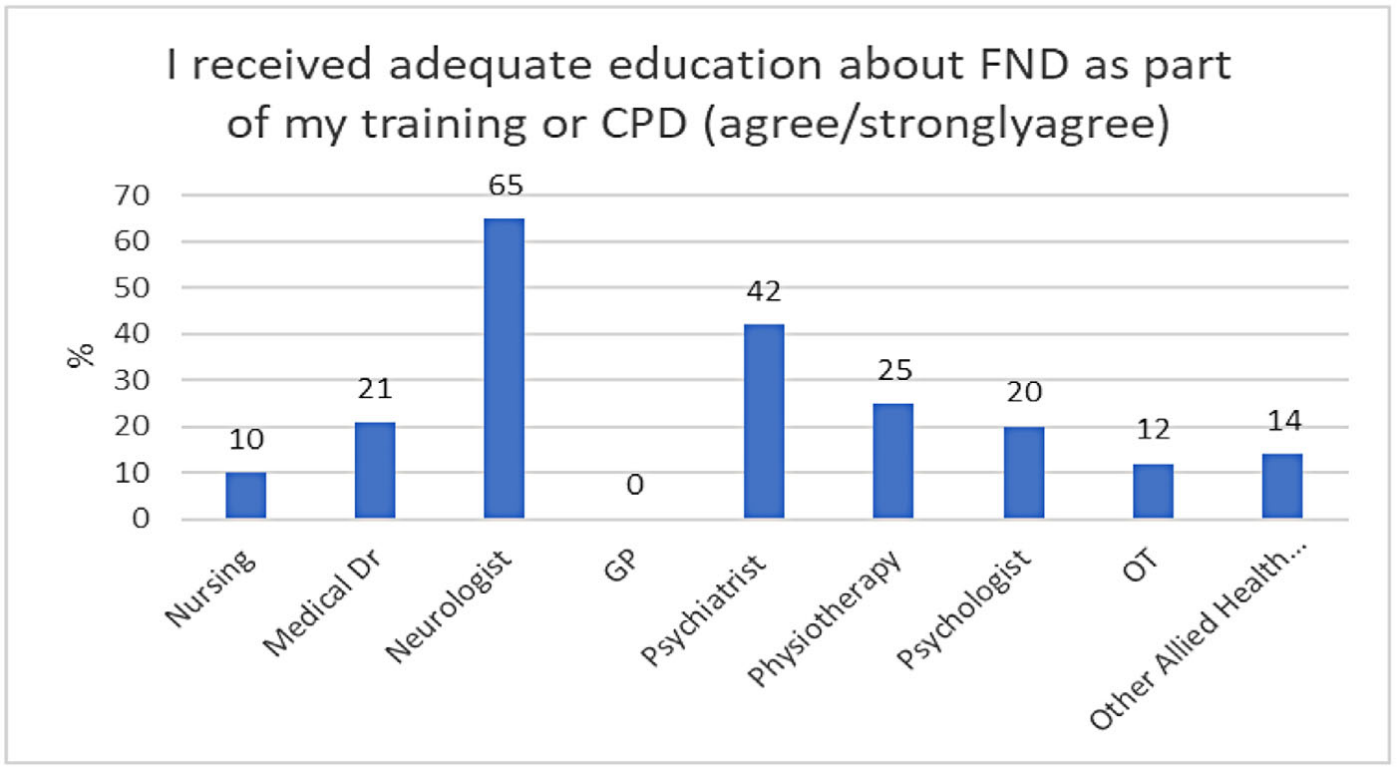
Perceived Level of Education about FND across profession.

Almost two‐thirds of respondents (63%) disagreed/strongly disagreed with the statement “I received adequate education about FNDs as part of my training or CPD.” (see Figure 3). While the majority of neurologists believed they had adequate training (65%), only a minority of each of the other disciplines agreed with this statement, including only 25.4% of all other medical professionals, despite 74% of them expressing an interest in learning more

The majority of survey respondents indicated that they would like to increase their knowledge about working with people with FND (89%).

Free‐text responses in relation to knowledge and training further supported the above findings, for example, *“knowledge of, training in and treatment of FND are at an embarrassing level for a modern medical system.”* Qualitative responses also highlighted the link between lack of knowledge of FND and negative attitudes toward patients: *“the level of misunderstanding (and sometimes overt disdain)…towards some patients needs to be addressed early through education and training.”*

## 4 DISCUSSION

This study was the first of its kind in Ireland exploring the perceptions of HCPs regarding patients with FND.  Previous international studies exploring attitudes of clinicians toward FND tended to focus predominantly on the perspective of medical professionals (Rawlings & Reuber, 2018), while this study aimed to capture the views of a range of disciplines, and is one of the largest multidisciplinary cohorts surveyed on this topic to date.

In terms of the challenges of dealing with patients with FND, over half of HCP respondents felt they required further specialist support to help these patients, with many highlighting the need for multidisciplinary input. Many respondents report low levels of support from referring HCPs, a finding also reported by Yogarajah et al. (2018).   Moreover, almost half of HCPs felt the referring doctor was not honest or open in terms of the referral. Poor communication between different health care providers has also been reported in other studies (Espay et al., 2018; Rawlings & Reuber, 2016; Warner et al., 2017). Many experienced some discomfort sharing the diagnosis, similar to previous research (Kanaan et al., 2011).

An additional challenge identified was the perceived lack of adequate time to manage these patients appropriately in clinic, a finding also noted by other studies (Warner et al., 2017). This challenge exists across both acute and community‐based health care settings. It is possible that this lack of clinic time reflects the fact that currently in Ireland there are no dedicated FND‐specific clinics in either the acute or community setting. Patients with FND are typically seen either in extremely busy general neurology clinics in acute hospitals or in general practices, where rushed consultations are likely to be unsatisfactory for clinician and patient alike. Patients with FND frequently report negative relationships with HCPs in Ireland and elsewhere (O'Keeffe et al., 2021; Rawlings & Reuber, 2016) and experience a sense of invalidation regarding symptoms (Foley et al., 2022). It is possible that these difficulties are amplified by insufficient clinic time.

Although the study respondents comprised a relatively experienced sample of HCPs (63% working in their current role for 11 years or more), the results revealed a lack of confidence in explaining a diagnosis of FND to patients among a number of disciplines. Only a slight majority of psychologists, neurologists and psychiatrists felt comfortable in this regard, despite all respondents having encountered these patients in clinic. A lack of confidence in diagnosing, managing and treating FND was revealed in over half of the studies reviewed by Phipps (Phipps, 2020).

Regarding attitudes toward FND, the survey results indicated that there continues to be a significant proportion of HCPs who find these patients “*difficult and demanding to deal with*.” This attitude is likely perpetuated by the reported lack of training and education opportunities and the perceived and/or actual lack of time available to properly address patient needs. Some respondents also acknowledged concerns about the validity of FND symptoms. In particular, 15% of HCPs endorsed the belief that these patients are malingering or faking, while a further 17% seemed unsure. These responses suggests uncertainty remains among some HCPs regarding FND symptom validity and again indicates a lack of understanding of the conditions aetiology. However, these proportions are less than those reported in previous studies, which were conducted primarily with medical professionals (Aatti et al., 2016; Ahern et al., 2009; Kanaan et al., 2011; Tinazzi et al., 2022; Yogarajah et al., 2018). Nonetheless, there is clearly still a need to improve this aspect of clinical knowledge and understanding in Ireland

The majority of respondents across all professions highlighted a significant lack of education about FND as part of their training and most expressed a strong interest in learning more about FND.  This is consistent with findings of other studies, which highlight a growing interest in this area in spite of inadequate education and training (Howman et al., 2016; Lehn et al., 2019; Yogarajah et al., 2018)

This data collected from Ireland informs the need for development and training for HCPs. It also provides a rich source of information on which recommendations for improved services for patients with FND can be developed. This is reflected by the finding that the vast majority of respondents believe patients with FND cannot access adequate service provision in Ireland. This may be linked to underfunding of appropriate services, reported poor communication between services, low patient contact and a lack of adequate time to support them.  Nevertheless, the findings suggest that greater awareness, interest, and educational input may be having a positive impact on perceptions in terms of FND symptom validity.

Given the high levels of interest expressed by HCPs in this study and the previously reported link between effective communication of the diagnosis of FND and positive clinical outcomes (Perjoc et al., 2023), efforts to increase education resources and investment in training of clinicians should be supported. Improved core training can impact clinician perceptions, the referral processes, and access to best practice treatments (Barnett et al., 2022), all of which is likely to be of clinical value to the patients with FND and their families. Furthermore, further education and investment in training is likely to be of economic value in terms of reduced acute health service utilization (Seneviratne et al., 2019).

Despite some positive developments in terms of guidance regarding the composition and design of services and clinical pathways for people with FND (e.g., Scotland NHS; Health improvement Scotland: Stepped care for Functional neurological disorders, 2012), service provision remains inadequate both in Ireland and elsewhere. This fragmented approach to care is further exacerbated by the lack of a cohesive strategy at an organisational level, which includes other disorders on the functional spectrum such as functional gastrointestinal symptoms and fibromyalgia, all of which could benefit from similar management and rehabilitative approaches.  The development of clinical guidelines and appropriately resourced FND clinical pathways in Ireland would be beneficial in enhancing the quality of life of people with FND (Goldstein, 2020; Perez et al., 2021).

### 4.1 Limitations

The survey targeted those with clinical interest in/involvement with patients with FND, which may limit the generalizability of findings to the wider population of HCPs in Ireland.  A sizable minority of respondents do not work frequently with these patients; that is, 40% of respondents see less than one FND patient per month. Using social media as a dissemination tool may target a larger proportion of younger, more motivated HCPs (Wasilewski et al., 2019).  The higher participation rate of physiotherapists in the study may also skew the sample findings toward the views of one profession. Most participants had been in their profession 11 years or more, reflecting a highly experienced cohort, perhaps not reflective of the national HCP population.  Finally, the smaller representation of some professions made it difficult to carry out inferential statistics, which limited the exploration of profession‐specific attitudes and training needs.

It nonetheless represents the first survey of its kind in relation to HCPs working with people with FND in Ireland. Future research could extend and expand the areas and items asked regarding attitudes and perceptions of FND, and examine a population‐wide cohort rather than those directly working with FND with an aim to understand wider perceptions throughout the healthcare system. Further qualitative studies of the experiences of HCPs would improve understanding of barriers to providing quality care, along with further examining the patients’ perspective of care, particularly as services and attitudes evolve.

## 5 CONCLUSIONS

This study aimed to understand the perceptions and attitudes of HCPs in Ireland toward FND. Overall, the findings indicated a significant need to continue to increase knowledge and training for HCPs regarding FND, in addition to supporting the development of appropriately resourced integrated, multidisciplinary FND care pathways, which allow adequate consultation length to comprehensively address care needs. Given the lack of clarity regarding reliability of numbers of patients of FND and accuracy of diagnosis, adequate training may help address this issue.

Placing a greater emphasis on education in FND in HCP training programmess from an undergraduate level along with supporting the development of clinical guidelines and structured service provision is now key to influencing change.

## AUTHOR CONTRIBUTIONS

**Roisin Vance**: conceptualization; investigation; writing—original draft; validation; methodology; writing—review and editing; project administration; resources; formal analysis. **Sarah Clarke**: writing—original draft; investigation; methodology; writing—review and editing; formal analysis; resources; software; data curation. **Fiadhnait O'Keefe**: writing—original draft; methodology; writing—review and editing; resources. **Toni Galligan**: software; formal analysis; writing—review and editing; data curation. **Anne Doherty**: writing—review and editing; investigation. **Cora Flynn**: investigation; writing—review and editing. **Eric Kelleher**: investigation; writing—review and editing. **Aoife Laffan**: writing—review and editing. **Colin Doherty**: writing—review and editing; investigation; methodology. **Diane Gillan**: investigation; writing—original draft; methodology; writing—review and editing; software; formal analysis; data curation; resources.

## CONFLICT OF INTEREST STATEMENT

The authors report no conflict of interest.

### PEER REVIEW

The peer review history for this article is available at https://publons.com/publon/10.1002/brb3.3362.

## Data Availability

The data that support the findings of this study are available from the corresponding author (RV) upon reasonable request.

## APPENDIX A DEMOGRAPHIC INFORMATION

(A)    DEMOGRAPHIC INFORMATION

Please answer the following questions about you and your practice

1. Sex

[] Male

[] Female

1. Age (years)

[ ] 18–24     [ ] 25–35      [ ] 36–45        [ ] 46–55        [ ] 56–65              [ ] > 66

1. Profession: ____________________
2. Years in practice in your current profession?

[] < 5 years       [] 6–10 years   [] 11–15 years  [] 16–20 years  [] > 21 years 

1. Which best describes the service you work in?

[ ] Hospital setting

[ ] Primary care setting

[ ] GP practice

[ ] Other ____________

1. Which best describes the number of patients with FND that you encounter per month?

[] < 1               [] 1–3                    [] 4–6                    [] 7–10         [] > 11

1. Do you currently work in a service in the Republic of Ireland? Yes ___  No  ___

## APPENDIX B SURVEY QUESTIONNAIRE

**Table:**

*Challenges to working with FND*	Strongly agree	Agree	Neither Agree nor Disagree	Disagree	Strongly Disagree
I don't have enough time to deal with these patients appropriately in my clinic/office					
I don't think I can help these patients much without specialist support from a psychologist/psychiatrist/neurologist/physiotherapist—please circle appropriate HCP(s)					
I generally feel well supported by other health professionals who refer patients with FNDs to me					
People with FND have access to appropriate services to manage their FND in Ireland					
Generally I am comfortable explaining the diagnosis of an FND to patients					
When I see patients with FNDs I often find that the referring doctor was not honest/open with the patient					
***Attitudes toward patients with FND***					
I find these patients often demanding and difficult to deal with					
I am often worried that these patients are actually malingering/faking/feigning					
These patients’ symptoms are real.					
***Knowledge and training in relation to FND***					
I received adequate education about FNDs as part of my training or CPD					
I am interested in learning more about working with people with FND					
**Free‐text responses**					
In terms of the management of patients with FND, what would be the most appropriate area of specialism to refer to, in your opinion? Please list in the text box below.					
Are there any other comments you would like to add?					

## References

[brb33362-bib-0001] Aatti, Y. , Schwan, R. , Maillard, L. , McGonigal, A. , Micoulaud‐Franchi, J. A. , de Toffol, B. , El‐Hage, W. , & Hingray, C. (2016). A cross‐sectional survey on French psychiatrists' knowledge and perceptions of psychogenic nonepileptic seizures. Epilepsy & Behavior, 60, 21–26. 27176880 10.1016/j.yebeh.2016.04.023

[brb33362-bib-0002] Ahern, L. , Stone, J. , & Sharpe, M. C. (2009). Attitudes of neuroscience nurses toward patients with conversion symptoms. Psychosomatics, 50(4), 336–339. 10.1176/appi.psy.50.4.336 19687173

[brb33362-bib-0003] Barnett, C. , Davis, R. , Mitchell, C. , & Tyson, S. (2022). The vicious cycle of functional neurological disorders: A synthesis of healthcare professionals' views on working with patients with functional neurological disorder. Disability and Rehabilitation, 44(10), 1802–1811. 10.1080/09638288.2020.1822935 32970485

[brb33362-bib-0004] Begley, R. , Farrell, L. , Lyttle, N. , Alty, J. , Curran, D. , Williams, S. , & Graham, C. D. (2023). Clinicians' implicit and explicit attitudes about the legitimacy of functional neurological disorders correlate with referral decisions. British Journal of Health Psychology, 28(2), 604–618. 10.1111/bjhp.12643 36626907

[brb33362-bib-0005] Bennett, K. , Diamond, C. , Hoeritzauer, I. , Gardiner, P. , McWhirter, L. , Carson, A. , & Stone, J. (2021). A practical review of functional neurological disorder (FND) for the general physician. Clinical Medicine, 21(1), 28–36. 10.7861/clinmed.2020-0987 33479065 PMC7850207

[brb33362-bib-0006] Carson, A. , Stone, J. , Hibberd, C. , Murray, G. , Duncan, R. , Coleman, R. , Warlow, C. , Roberts, R. , Pelosi, A. , Cavanagh, J. , Matthews, K. , Goldbeck, R. , Hansen, C. , & Sharpe, M. (2011). Disability, distress and unemployment in neurology outpatients with symptoms ‘unexplained by organic disease’. Journal of Neurology, Neurosurgery, and Psychiatry, 82(7), 810–813. 10.1136/jnnp.2010.220640 21257981

[brb33362-bib-0007] Ducroizet, A. , Zimianti, I. , Golder, D. , Hearne, K. , Edwards, M. , Nielsen, G. , & Coebergh, J. (2023). Functional neurological disorder: Clinical manifestations and comorbidities: an online survey. Journal of Clinical Neuroscience, 110, 116–125. 10.1016/j.jocn.2023.02.014 36871491

[brb33362-bib-0008] Espay, A. J. , Aybek, S. , Carson, A. , Edwards, M. J. , Goldstein, L. H. , Hallett, M. , LaFaver, K. , LaFrance, W. C., Jr , Lang, A. E. , Nicholson, T. , Nielsen, G. , Reuber, M. , Voon, V. , Stone, J. , & Morgante, F. (2018). Current concepts in diagnosis and treatment of functional neurological disorders. JAMA Neurology, 75(9), 1132–1141. 10.1001/jamaneurol.2018.1264 29868890 PMC7293766

[brb33362-bib-0009] Fobian, A. D. , & Elliott, L. (2019). A review of functional neurological symptom disorder etiology and the integrated etiological summary model. Journal of Psychiatry & Neuroscience, 44(1), 8–18. 30565902 10.1503/jpn.170190 PMC6306282

[brb33362-bib-0010] Foley, C. , Kirkby, A. , & Eccles, F. J. R. (2022). A meta‐ethnographic synthesis of the experiences of stigma amongst people with functional neurological disorder. Disability and Rehabilitation, 1–12. Advance online publication. 10.1080/09638288.2022.2155714 36519449

[brb33362-bib-0011] Gelauff, J. , Stone, J. , Edwards, M. , & Carson, A. (2014). The prognosis of functional (psychogenic) motor symptoms: A systematic review. Journal of Neurology, Neurosurgery, and Psychiatry, 85(2), 220–226. 10.1136/jnnp-2013-305321 24029543

[brb33362-bib-0012] Goldstein, L. H. , Robinson, E. J. , Mellers, J. D. C. , Stone, J. , Carson, A. , Reuber, M. , Medford, N. , McCrone, P. , Murray, J. , Richardson, M. P. , Pilecka, I. , Eastwood, C. , Moore, M. , Mosweu, I. , Perdue, I. , Landau, S. , & Chalder, T. , CODES study group . (2020). Cognitive behavioural therapy for adults with dissociative seizures (CODES): A pragmatic, multicentre, randomised controlled trial. The Lancet Psychiatry, 7(6), 491–505. 32445688 10.1016/S2215-0366(20)30128-0 PMC7242906

[brb33362-bib-0013] Hallett, M. , Aybek, S. , Dworetzky, B. A. , McWhirter, L. , Staab, J. P. , & Stone, J. (2022). Functional neurological disorder: New subtypes and shared mechanisms. The Lancet. Neurology, 21(6), 537–550. 10.1016/S1474-4422(21)00422-1 35430029 PMC9107510

[brb33362-bib-0014] Health improvement Scotland: Stepped care for Functional neurological disorders . (2012). www.healthcareimprovementscotland.org

[brb33362-bib-0015] Howman, M. , Walters, K. , Rosenthal, J. , Ajjawi, R. , & Buszewicz, M. (2016). You kind of want to fix it don't you? Exploring general practice trainees' experiences of managing patients with medically unexplained symptoms. BMC Medical Education [Electronic Resource], 25, 16–27. 10.1186/s12909-015-0523-y PMC4727318 26810389

[brb33362-bib-0016] Kanaan, R. A. , Armstrong, D. , & Wessely, S. C. (2011). Neurologists' understanding and management of conversion disorder. Journal of Neurology, Neurosurgery, and Psychiatry, 82(9), 961–966. 10.1136/jnnp.2010.233114 21325661 PMC3191819

[brb33362-bib-0017] Lehn, A. , Bullock‐Saxton, J. , Newcombe, P. , Carson, A. , & Stone, J. (2019). Survey of the perceptions of health practitioners regarding functional neurological disorders in Australia. Journal of Clinical Neuroscience: Official Journal of the Neurosurgical Society of Australasia, 67, 114–123. 10.1016/j.jocn.2019.06.008 31229424

[brb33362-bib-0018] O'Keeffe, S. , Chowdhury, I. , Sinanaj, A. , Ewang, I. , Blain, C. , Teodoro, T. , Edwards, M. , & Yogarajah, M. (2021). A service evaluation of the experiences of patients with functional neurological disorders within the NHS. Frontiers in Neurology, 12, 656466. 10.3389/fneur.2021.656466 34135848 PMC8200476

[brb33362-bib-0019] Perez, D. L. , Edwards, M. J. , Nielsen, G. , Kozlowska, K. , Hallett, M. , & LaFrance, W. C., Jr (2021). Decade of progress in motor functional neurological disorder: Continuing the momentum. Journal of Neurology, Neurosurgery, and Psychiatry, 2021; jnnp‐2020‐323953. Advance online publication. 10.1136/jnnp-2020-323953 PMC8440656 33722822

[brb33362-bib-0020] Perjoc, R. S. , Roza, E. , Vladacenco, O. A. , Teleanu, D. M. , Neacsu, R. , & Teleanu, R. I. (2023). Functional neurological disorder‐old problem new perspective. International Journal of Environmental Research and Public Health, 20(2), 1099. 10.3390/ijerph20021099 36673871 PMC9859618

[brb33362-bib-0021] Phipps, K. M. (2020). An exploration of factors influencing healthcare professionals’ perceptions towards functional neurological disorders (p. 131). Doctoral Thesis, University of Southampton. http://eprints.soton.ac.uk/id/eprint/446917

[brb33362-bib-0022] Rawlings, G. H. , & Reuber, M. (2016). What patients say about living with psychogenic nonepileptic seizures: A systematic synthesis of qualitative studies. Seizure: The Journal of the British Epilepsy Association, 41, 100–111. 10.1016/j.seizure.2016.07.014 27522576

[brb33362-bib-0023] Rawlings, G. H. , & Reuber, M. (2018). Health care practitioners’ perceptions of psychogenic nonepileptic seizures: A systematic review of qualitative and quantitative studies. Epilepsia, 59(6), 1109–1123. 10.1111/epi.14189 29750340

[brb33362-bib-0024] Seneviratne, U. , Low, Z. M. , Low, Z. X. , Hehir, A. , Parameswaran, S. , Foong, M. , Ma, H. , & Phan, T. G. (2019). Medical health care utilization cost of patients presenting with psychogenic nonepileptic seizures. Epilepsia, 60(2), 349–357. 10.1111/epi.14625 30577087

[brb33362-bib-0025] Stephen, C. D. , Fung, V. , Lungu, C. I. , & Espay, A. J. (2021). Assessment of emergency department and inpatient use and costs in adult and pediatric functional neurological disorders. JAMA Neurology, 78(1), 88–101. 10.1001/jamaneurol.2020.3753 33104173 PMC7589058

[brb33362-bib-0026] Stone, J. , Burton, C. , & Carson, A. (2020). Recognising and explaining functional neurological disorder. BMJ (Clinical Research Ed.), 371, m3745. 10.1136/bmj.m3745 33087335

[brb33362-bib-0027] Stone, J. , Warlow, C. , & Sharpe, M. (2010). The symptom of functional weakness: A controlled study of 107 patients. Brain, 133, 1537–1551. 10.1093/brain/awq068 20395262

[brb33362-bib-0028] Tinazzi, M. , Fiorio, M. , Berardelli, A. , Bonetti, B. , Bonifati, D. M. , Burlina, A. , Cagnin, A. , Calabria, F. , Corbetta, M. , Cortelli, P. , Giometto, B. , Guidoni, S. V. , Lopiano, L. , Mancardi, G. , Marchioretto, F. , Pellegrini, M. , Teatini, F. , Tedeschi, G. , Tesolin, L. , & Turinese, E. M. (2022). A. Opinion, knowledge, and clinical experience with functional neurological disorders among Italian neurologists: Results from an online survey. Journal of Neurology, 269(5), 2549–2559. 10.1007/s00415-021-10840-y 34665330 PMC9021063

[brb33362-bib-0029] Warner, A. , Walters, K. , Lamahewa, K. , & Buszewicz, M. (2017). How do hospital doctors manage patients with medically unexplained symptoms: A qualitative study of physicians. Journal of the Royal Society of Medicine, 110(2), 65–72. 10.1177/0141076816686348 28169588 PMC5305010

[brb33362-bib-0030] Wasilewski, M. B. , Stinson, J. N. , Webster, F. , & Cameron, J. I. (2019). Using Twitter to recruit participants for health research: An example from a caregiving study. Health Informatics Journal, 25(4), 1485–1497. 10.1177/1460458218775158 29843545

[brb33362-bib-0031] Yogarajah, M. , Child, R. , Agrawal, N. , Cope, S. , Edwards, M. , & Mula, M. (2018). Functional seizures: An evaluation of the attitudes of general practitioners local to a tertiary neuroscience service in London. Epilepsia Open, 19(1), ;4 54–62. 10.1002/epi4.12283 PMC6398091 30868115

